# The decoupling between genetic structure and metabolic phenotypes in *Escherichia coli* leads to continuous phenotypic diversity

**DOI:** 10.1111/j.1420-9101.2011.02287.x

**Published:** 2011-07

**Authors:** V Sabarly, O Bouvet, J Glodt, O Clermont, D Skurnik, L Diancourt, D de Vienne, E Denamur, C Dillmann

**Affiliations:** * DGA/CNRS, UMR de Génétique Végétale INRA/CNRS/Univ Paris-Sud, Ferme du MoulonGif-sur-Yvette, France; † INSERM U722 and Université Paris-Diderot, Faculté de Médecine, Site Xavier BichatParis, France; ‡ Univ Paris-Sud, UMR de Génétique Végétale INRA/CNRS/Univ Paris-Sud, Ferme du MoulonGif-sur-Yvette, France; § Genotyping of Pathogens and Public Health, Institut PasteurParis, France

**Keywords:** carbon source, *E. coli*, genetic distance, genetic group, lifestyle, metabolic pathway, natural isolate, phenotypic distance

## Abstract

To assess the extent of intra-species diversity and the links between phylogeny, lifestyle (habitat and pathogenicity) and phenotype, we assayed the growth yield on 95 carbon sources of 168 *Escherichia* strains. We also correlated the growth capacities of 14 *E. coli* strains with the presence/absence of enzyme-coding genes. Globally, we found that the genetic distance, based on multilocus sequence typing data, was a weak indicator of the metabolic phenotypic distance. Besides, lifestyle and phylogroup had almost no impact on the growth yield of non-*Shigella E. coli* strains. In these strains, the presence/absence of the metabolic pathways, which was linked to the phylogeny, explained most of the growth capacities. However, few discrepancies blurred the link between metabolic phenotypic distance and metabolic pathway distance. This study shows that a prokaryotic species structured into well-defined genetic and lifestyle groups can yet exhibit continuous phenotypic diversity, possibly caused by gene regulatory effects.

## Introduction

Species have been first differentiated from morphological traits, and nowadays phenotypic criteria are still used to characterize them. Even for bacteria, phenotypic characteristics should agree with phylogenetic relatedness to constitute a species (Wayne *et al.*, 1987; Stackebrandt *et al.*, 2002). The underlying idea is that genetically distinct organisms should also be phenotypically distinct. Several cases, for which phylogeny, phenotype and ecological niche are related, support this view. For instance, in the group of asexual species of bdelloid rotifers, genetic and morphological clusters are the same and result from niche divergence (Fontaneto *et al.*, 2007). In bacteria of the genus *Bacillus*, genetic groups and growth temperature are also linked as a consequence of the ecology of these species (Guinebretière *et al.*, 2008).

However, several studies have revealed that the genetic distances and the phenotypes can be poorly related, as it has been found in eukaryotes species such as *Zea mays* (maize) (Burstin & Charcosset, 1997) and *Lolium perenne* (ryegrass) (Roldán-Ruiz *et al.*, 2001). Similar results have been observed for bacterial species such as members of the genus *Cronobacter* (Baldwin *et al.*, 2009) and strains of *Staphylococcus aureus* (Morandi *et al.*, 2010). A well-known phenomenon that can disrupt the link between genetic distance and phenotype is the phenotypic convergence resulting from similar ecological niches of distinct genetic groups. For instance, life-history strategies are associated with specific habitats in *Saccharomyces cerevisiae*, and genetically distant strains sharing the same habitat have similar life-history strategies (Spor *et al.*, 2009).

The *E. coli* species is of particular interest to study the relationships between phylogenetic relatedness and phenotypic variation. The evolutionary history of the species (Lecointre *et al.*, 1998) revealed that the strains are distributed among five main phylogroups: A, B1, B2, D and E (Herzer *et al.*, 1990; Escobar-Páramo *et al.*, 2004a). In addition, natural isolates of *E. coli* are found in a variety of habitats, which can be either vertebrate hosts or water or soil (Hartl & Dykhuizen, 1984) and can be commensals (Tenaillon *et al.*, 2010), intra-intestinal pathogens (Intestinal Pathogenic *E. coli* or InPEC) or extra-intestinal pathogens (extra-intestinal pathogenic *E. coli* or ExPEC) (Kaper *et al.*, 2004). We chose to call lifestyles the combinations of habitat and pathogenicity. The prevalence of the different phylogroups varies slightly between lifestyles. For instance, farm animals exhibit a higher proportion of A and B1 strains and a lower proportion of B2 and D strains than wild animals. Likewise, ExPEC strains belong mainly to the phylogroup B2 (Picard *et al.*, 1999). However, there is no clear-cut link between phylogroups and lifestyles, i.e. no lifestyle can be uniquely attributed to a given phylogroup (Gordon & Cowling, 2003; Escobar-Páramo *et al.*, 2006). *E. coli* genome, which encompasses approximately 4700 genes, is highly dynamic: the core-genome, the genes present in all the sequenced genomes, is about 2000 genes, whereas the pan genome, the full set of nonorthologous genes among all genomes, reaches 18 000 genes (Rasko *et al.*, 2008; Touchon *et al.*, 2009).

Based on this large genetic diversity and the various lifestyles, we expect to find a large phenotypic variation within the species. The nonrandom distribution of the phylogroups among different lifestyles may indicate that these groups differ in phenotypes. Besides, as anthropogenic factors such as domestication play a major role in the ecological structure and the level of antimicrobial resistance of *E. coli* (Escobar-Páramo *et al.*, 2006; Skurnik *et al.*, 2006), the exposure of a strain animal host to humans could influence the phenotype of the bacterium. The prevalence of *E. coli* and the relative abundance of the phylogroups depend on the host diet (Gordon & Cowling, 2003), which might also have an impact on the strain phenotype. Finally, the strain phenotype could be globally linked to the pathogenic nature of the bacterium as this has been shown to be the case for a given metabolic phenotypic character. Indeed, the use of deoxyribose constitutes a fitness advantage for the competitiveness of extra-intestinal pathogenic *E. coli* strains (Bernier-Febreau *et al.*, 2004; Martinez-Jéhanne *et al.*, 2009).

To assess the extent of intra-species diversity as well as the links between phylogeny, lifestyle and phenotype, we assayed the growth yield (carbon source utilization) of a panel of genetically diverse *E. coli* natural isolates. We included several phylogenetic outgroups in the study as well as one phenotypic outgroup to test whether our methodology gives a global and representative image of a strain phenotype. Metabolic capacities are conditioned by the occurrence of specific enzymatic reactions in the cell that can be inferred from the strain gene content. Therefore, to go further, we studied in a subset of strains the relationship between growth capacities and metabolic pathways reconstructed from complete genome data. Hence, we were able to analyse the correlations between phylogenetic distance, metabolic phenotypic distance and metabolic pathway presence. Overall, the strain growth yield seemed to present continuous variations around the species average, whereas the pattern of the presence/absence of the metabolic pathways was linked to the species phylogeny. Finally, we discussed the impact of the species life cycle on the metabolic phenotypic diversity and the molecular mechanisms that could account for discrepancies between growth and the presence of metabolic pathways.

## Materials and methods

### Bacterial strains

The growth experiments were conducted on 168 bacterial strains comprising 159 *E. coli*/*Shigella* strains, six cryptic *Escherichia* clade strains, two *E. fergusonii* strains and one *E. albertii* strain. *E. fergusonii*, *E. albertii* and cryptic *Escherichia* clade strains were used as phylogenetic outgroups. The cryptic *Escherichia* clades are *Escherichia* lineages that have recently been reported. Strains belonging to these clades are very divergent from *E. coli* based on DNA sequence data; however, no biochemical feature allowed distinguishing them from *E. coli* (Walk *et al.*, 2009). The non-*Shigella E. coli* strains were chosen as representative of the genetic diversity of the species based on the triplex PCR phylogrouping (Clermont *et al.*, 2000) and multilocus sequence typing (MLST) data from more than 4000 isolates from various collections (Picard *et al.*, 1999; Escobar-Páramo *et al.*, 2004a,b, 2006; Clermont *et al.*, 2011). To have four groups of comparable genetic diversity, we chose to make one genetic group, A/B1, from the close A and B1 phylogroups. One hundred and fifty *E. coli* strains belonged to the genetic groups A/B1 (75 strains), B2 (38 strains), D (26 strains) and E (11 strains). Moreover, three strains did not belong to any group and were thus labelled ‘ungrouped’. We also included six *Shigella* strains distributed into different *Shigella*-specific phylogroups (two in S1, one in S2, one in S3, one in SD1 and one in SS [Pupo *et al.*, 2000; Escobar-Páramo *et al.*, 2003]). These strains were used as phenotypic outgroup. Indeed, *Shigella* strains are intra-cellular human-specific pathogens that emerged from different *E. coli* phylogroups but present similar distinctive biochemical features as a consequence of their common lifestyle (Pupo *et al.*, 2000; Escobar-Páramo *et al.*, 2003). The 153 non-*Shigella E. coli* strains were divided into several lifestyle groups: three pathogenic groups (commensal [90 strains], ExPEC [28 strains] and InPEC [35 strains]), four host anthropogenic groups (according to their exposure to humans: humans [53 strains], pet dogs [19 strains], farm animals [49 strains] and wildlife animals [32 strains]) (Skurnik *et al.*, 2006) and four host diets (insectivorous and granivorous birds [19 strains], carnivorous mammals [24 strains], herbivorous mammals [39 strains] and omnivorous mammals [71 strains]). The strains were selected to have comparable genetic diversity in the different lifestyle groups. The study on the relationship between growth capacities and metabolic pathways was conducted on a subset of 13 commensal and pathogenic *E. coli* strains for which the complete genome sequence was available (http://www.genoscope.cns.fr/agc/microscope/) as well as on the laboratory strain K-12. The main characteristics of all the strains are given Table S1. For each strain, the reference stock was conserved at −80 °C with glycerol.

### Growth assays

Cells from the stock were grown overnight in Luria–Bertani broth at 37 °C then pelleted and washed once with minimal buffer (100 mm NaCl, 30 mm triethanolamine HCl, 5 mm NH_4_Cl, 2 mm NaH_2_PO_4_, 0.25 mm Na_2_SO_4_, 0.05 mm MgCl_2_, 1 mm KCl, 1 μm FeCl_3_ and pH 7.1) and finally resuspended in minimal buffer. Growth capacities were assayed using commercially available Biolog GN2 microplates (AES Chemunex, Combourg, France). Each of the 96 wells of a Biolog GN2 microplate contains a simple carbon source presented Fig. S1, except one used as control, and a tetrazolium dye, which is an indicator of oxidative carbon metabolism correlated with bacterial growth (MacLean & Bell, 2002, 2003; MacLean *et al.*, 2004; Venail *et al.*, 2008). Each well of the Biolog GN2 microplates was inoculated with 100 μL of cell suspension diluted at an optical density (OD) of 0.03 measured on an Ultrospec 1100 pro spectrophotometer.

We measured the OD at 750 nm with a Tecan Infinite M200 plate reader after 18 h of growth at 37 °C in an incubator where the plates were shaken. We then subtracted the blank value (OD reached in the control well) to the OD after culture in each well. We called this value the growth yield. Experiments were conducted at eight different dates with a block design for the strains and 14 strains were replicated twice. Growth yield was corrected for a date effect using its least-square mean value computed by an analysis of variance (anova) comprising five factors: the date of the assay, the phylogenetic group of the strain, its pathogenic group, the strain host anthropogenic group and its diet. The residual of the model contained both experimental error and genetic variation between strains of the same group. A separate analysis was conducted for each carbon source (see section Statistical analyses). All subsequent analyses were performed on growth yield corrected for the date effect. To determine a threshold above which growth was considered to be positive, we applied Gaussian mixture models to the growth yields (Fraley & Raftery, 2002, 2006). The optimal model according to the Bayesian information criterion (BIC) had three components: two of them with an average OD close to zero and the third one with an average OD close to one (Fig. S2). We chose to consider growth as positive whenever the corrected OD belonged to the third population with a 5% false-positive rate. Hence, positive growth corresponded to a growth yield >0.3388 OD units.

### Phylogeny and genetic divergence

To estimate the genetic divergence between strains, we used the MLST data generated from eight partial genes: *din*B (450 bp), *icd*A (516 bp), *pab*B (468 bp), *pol*B (450 bp), *put*P (456 bp), *trp*A (561 bp), *trp*B (594 bp) and *uid*A (600 bp) (Jaureguy *et al.*, 2008; http://www.pasteur.fr/recherche/genopole/PF8/mlst/EColi.html). The phylogenetic tree was inferred with the software PhyML 3.0 (Guindon & Gascuel, 2003) using a generalized time-reversible (GTR) model with optimized equilibrium frequencies, estimated proportion of invariable sites, four substitution rate categories using the mean as the centre of each class and estimated gamma distribution parameter. The tree topology was optimized to maximize the likelihood using the nearest neighbour interchanges (NNIs) tree topology search operation with no random starting tree and a neighbour-joining input tree. The tree was plotted with the R package APE (Paradis *et al.*, 2004). The genetic divergence (*d*_G_) is the distance between strains derived from this phylogenetic tree by the R package APE.

### Statistical analyses

Analyses of variance were performed on each substrate for which growth was positive for at least one strain. The growth yield was analysed using a linear model with four main effects: the phylogenetic group of the strain, its pathogenic group, the strain host anthropogenic group and its diet. Type-III anova tables were computed using the R package car (Fox & Weisberg, 2010). *P*-values of the *F*-tests from the anova tables were cumulated, and the effects for which the false-positive discovery rate, FDR (Benjamini & Hochberg, 1995; Strimmer, 2009), was <0.1 % were considered significant. For those effects, we computed the least-square means and corresponding error variance for each group. The growth yield was also used to determine the metabolic phenotypic distance (*d*_P_) between strains, the Euclidean distance between vectors of growth yields and to run a principal component analysis (PCA) computed with the software R (R Development Core Team, 2009). All the Mantel tests between the different distances were performed using the R package ade4 (Dray & Dufour, 2007).

### Metabolic pathways

In the study on the relationship between growth capacities and metabolic pathways, which comprised fewer strains (14 strains for which the complete genome sequence was available), the growth assay procedure was the same than for the other growth experiments except that the OD at 750 nm of each well of the microplates were monitored every 25 min during the 18 h of growth at 37 °C in a Tecan Infinite M200 plate reader where the plates were also shaken. The whole process (overnight growth and microplating) was repeated at least twice on different days. Thus, for each carbon source, growth was represented by two to three curves. The growth yield was estimated from the growth curve after performing a cubic spline interpolation using the software R (R Development Core Team, 2009). It corresponded to the amplitude of growth, i.e. to the OD reached after 18 h of growth minus the initial OD. This procedure minimized the biochemical assay errors to compare the metabolic capabilities of the strains with their gene contents. Growth was considered positive if the growth yield, averaged on the replicates, was greater than the growth threshold (0.2578 OD units), determined using Gaussian mixture models as in the other growth experiments.

The metabolic pathways present in the sequenced strains were recovered using the metabolic profiles from the Microcyc website (http://www.genoscope.cns.fr/agc/microcyc). The process to determine these metabolic profiles is as described in Vieira *et al.*, 2011. For one strain, each pathway was represented by its completion percentage. For example, a pathway for which all the enzyme-coding genes are present in the genome has a completion of 1, if half the enzyme-coding genes are missing, the completion is 0.5, and 0 if the pathway was not inferred in the strain. We defined the metabolic pathway completion distance between two strains (*d*_M_) as the Euclidean distance between their vectors of pathway completions. To link the carbon sources allowing growth of at least one of the 14 sequenced strains to the metabolic pathways specifically involved in their degradation, we first selected all the pathways where the carbon source intervened as substrate or product of a reaction. Then, among this first selection of pathways, we manually removed those not involved in the degradation of the carbon source of interest. To link a maximum of carbon sources to pathways, we manually added four pathways because they involved reactions not classified as part of a pathway or because the reactions were not described yet in the Metacyc 13.0 database. These pathways concerned the following substrates: *N*-acetyl-d-galactosamine (enzymes: N-acetylglucosamine-6-phosphate deacetylase, EC 3.5.1.25; galactosamine-6-phosphate isomerase, no EC; 6-phosphofructokinase I, EC 2.7.1.11; tagatose 6-phosphate aldolase 1, EC 4.1.2.40 [Mukherjee *et al.*, 2008]), lactulose (enzyme: cryptic beta-D-galactosidase, EC 3.2.1.23), D-serine (enzyme: D-serine ammonia-lyase, EC 4.3.1.18) and D-raffinose (enzyme: alpha-galactosidase, EC 3.2.1.22). We successfully matched 43 carbon sources to their degradation pathways (Table S2) but we were unable to relate the consumption of glycyl-L-aspartic acid, L-alanyl-glycine and methylpyruvate to any metabolic pathway.

### Phenotypic and metabolic distance models

We implemented a simplified model for the relationship between the metabolic phenotypic distance (*d*_P_) between two strains and their genetic distance (*d*_G_), defined here as the proportion of genes that are not identical by descent between the two strains. Some of the genetic differences can also be because of horizontal gene transfers independently of their phylogeny with a probability *Λ*. Moreover, only a fraction of the genetic differences cause gene inactivation. We called *μ* the probability that a genetic difference did not change the gene functionality. Therefore, the probability *p*_M_ that two genes had a functional difference was
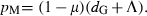
(1)

Genetic differences may not always translate into phenotypic differences. Here, the phenotypic observation is the growth (*P*= 1) or absence of growth (*P*= 0) on a given carbon source. We supposed that all *n* genes of the pathway needed to be functional for the pathway to be functional (*M* = 1). Hence, the probability that two strains had a functional difference (Δ*M* ≠ 0) for a given carbon source was
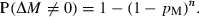
(2)

Our lack of knowledge on the metabolic network as well as differences in the gene regulatory network can lead to unexpected phenotypes according to the pathway functionalities. We defined the parameter *δ*_M_ as the probability that two strains share a common phenotype (Δ*P* = 0) on a given substrate while having different pathway functionalities (Δ*M* ≠ 0) concerning this carbon source: *δ*_M_ = P(Δ*P* = 0 | Δ*M* ≠ 0). Similarly, *δ*_P_ was the probability that two strains have different phenotypes (Δ*P* ≠ 0) while having the same pathway functionalities (Δ*M* = 0): *δ*_P_ = P(Δ*P* ≠ 0 | Δ*M* = 0). Thus, the probability that two strains have different growth capacities on a carbon source was
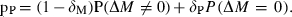
(3)

Monte Carlo simulations were performed to assess the relationship between genetic and phenotypic distances using parameters taken from our experimental data. We used the number of genes implied in each of the 395 metabolic pathways recovered in the 14 sequenced strains we studied. The proportion of genes that are not identical between two strains is proportional to the genetic distance between these strains and were consequently drawn in an uniform distribution on an interval corresponding to the observed values for our data (between zero and 0.25). We computed *p*_M_ between 10 000 strain pairs with *μ*= 0.83 (Patel & Loeb, 2000) and *Λ*= 0.13 (Ochman *et al.*, 2000). For each pathway, the number of genes having different functionalities between two strains was drawn in a binomial distribution with a probability *p*_M_ and a number of trials equal the number of genes implied in the pathway. The metabolic pathway completion distance between two strains (*d*_M_), defined as the Euclidean distance between vectors of pathway completions, was then calculated, as well as the vector of differences for pathway functionalities Δ*M*. The metabolic phenotypic distance between two strains (*d*_P_), defined as the Euclidean distance between vectors of qualitative growth status, was computed as the square root of the sum of two random variables following binomial laws: the first one of probability 1 − *δ*_M_ on all the carbon sources for which the pathway functionalities differed between the two strains (Δ*M* ≠ 0), and the second one of probability *δ*_P_ on all the carbon sources corresponding to pathways having the same functionality (Δ*M* = 0) (eqn 3). We determined *δ*_M_ and *δ*_P_ using the metabolic pathway completion and growth data for each strain couple of the 14 sequenced strains and took the average values as estimates: *δ*_M_ = 0.63 and *δ*_P_ = 0.21. Moderate changes of the parameter values (*μ*, *Λ*, *δ*_M_ and *δ*_P_) did not significantly change the simulation output (data not shown).

## Results

### The genetic distance is a weak indicator of the metabolic phenotypic distance

**Fig. 1:**
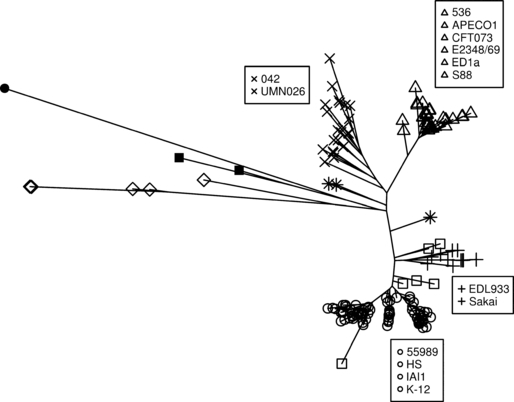
Phylogenetic tree of 169 *Escherichia* strains reconstructed from the partial sequences of eight housekeeping genes by maximum likelihood. Non-*Shigella E. coli* strains are divided into four genetic groups, A/B1 (o), B2 (Δ), D (×) and E (+), as well as into an ungrouped category (*). The *Shigella* strains, although not monophyletic, are considered as a specific group represented by empty squares (□); they belong to particular phylogroups (S1, S2, S3, SD1 and SS). Cryptic *Escherichia* clade strains are indicated by diamonds (⋄), *E. fergusonii* strains by filled squares (▪) and the *E. albertii* strain by a filled circle (•). The names of the 14 sequenced strains used in the metabolic pathway study are given in the boxes. This phylogeny is in agreement with the one obtained using complete genome sequences (Touchon *et al.*, 2009).

**Fig. 2:**
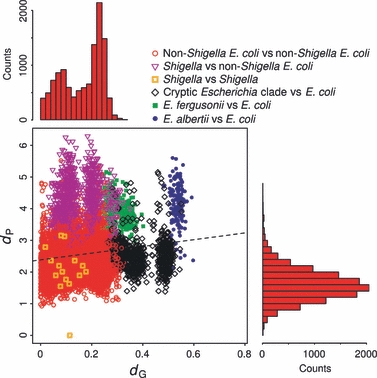
Relationship between the metabolic phenotypic distance, *d*_P_, and the genetic distance, *d*_G_, resulting from comparisons between 159 *E. coli* strains (comprising six *Shigella* strains), six cryptic *Escherichia* clade strains, two *E. fergusonii* strains and one *E. albertii* strain. Only the comparisons involving at least one *E. coli* strain were considered. The dashed line corresponds to the regression of *d*_P_ according to *d*_G_ taking into account all represented strain pairs. The histograms represent the distributions of *d*_G_ (on top) and *d*_P_ (on the right) for the comparisons between two non-*Shigella E. coli* strains corresponding to the red circles on the plot.

Our strain sample consisted in 159 *E. coli* strains (comprising six *Shigella* strains), six cryptic *Escherichia* clade strains, two *E. fergusonii* strains and one *E. albertii* strain. The phylogenetic tree of these strains shows that the non-*Shigella E. coli* strains constitute four distinct genetic groups (A/B1, B2, D and E) (Fig. 1). To estimate their metabolic phenotypic diversity, we assessed their growth yield on 95 different carbon sources. Figure 2 represents the plot of the metabolic phenotypic distance (*d*_P_) vs. the genetic distance (*d*_G_) between couples of strains. As expected, *E. fergusonii* strains as well as *E. albertii* strains are clearly distant both genetically and phenotypically, whereas cryptic *Escherichia* clade strains are quite divergent genetically but not phenotypically. That is why they have only recently been uncovered although they are genetically very divergent from *E. coli* (Walk *et al.*, 2009). On the contrary, *Shigella* strains, which show *d*_G_ of the same order than other couples of *E. coli* strains, are phenotypically distinct when compared to non-*Shigella E. coli* strains. However, two *Shigella* strains present *d*_P_ similar to the ones between two non-*Shigella E. coli* strains, which confirms the phenotypic convergence of these strains. Therefore, our phenotypic assay allows for a representative determination of a strain global phenotype.

Overall, there was only a very weak correlation between *d*_G_ and *d*_P_ (Mantel test *R*² = 0.02, *P*-value = 0.0076). When the *Shigella* and cryptic *Escherichia* clade strains were removed, the correlation increased (Mantel test *R*² = 0.10, *P*-value < 0.0001), showing that these two opposite cases (low *d*_G_, high *d*_P_ and high *d*_G_, low *d*_P_) are typical causes of the disruption of the link between *d*_G_ and *d*_P_. Within the non-*Shigella E. coli* strains, the correlation is still significant but very weak (Mantel test *R*² = 0.01, *P*-value = 0.0036). Interestingly, the distribution of *d*_G_ for the non-*Shigella E. coli* strains exhibited two peaks corresponding to the intra-phylogroup and inter-phylogroup comparisons, whereas the distribution of *d*_P_ was unimodal (Fig. 2). Thus, although *E. fergusonii* and *E. albertii* species as well as *Shigella* strains appeared clearly distinct phenotypically from non-*Shigella E. coli* strains, the different *E. coli* phylogroups rather seemed to display continuous phenotypic variations. The structure of the metabolic phenotypic diversity within *E. coli* species is unknown, and thus, in the following analyses, we focused on non-*Shigella E. coli* strain metabolic phenotypes in relation to the phylogroups and lifestyles of these strains.

### Most growth yield variation is independent from the strain phylogeny and lifestyle

**Fig. 3:**
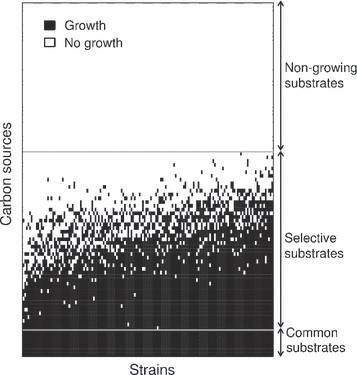
Diversity of carbon source use by 153 non-*Shigella Escherichia coli* strains. Seven carbon sources allowed the growth of all the 153 strains (common substrates) and 48 of only a fraction of them (selective substrates), whereas 40 did not allow any growth (nongrowing substrates). The strains are ordered by the number of substrates they can catabolize. The carbon sources are ordered by the number of strains able to grow on them. See Fig. S1 for a detailed version.

**Fig. 4:**
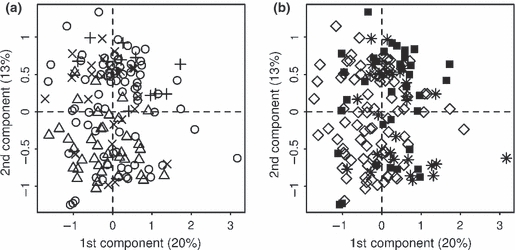
Principal component analysis (PCA) of 150 non-*Shigella Escherichia coli* strains based on their growth yield on 95 carbon sources. In (A) the symbols correspond to the phylogroups: A/B1 (o), B2 (Δ), D (×) and E (+) (the three ungrouped strains were discarded). In (B) the symbols correspond to the pathogenic groups: commensal (⋄), ExPEC (*), InPEC (▪). Percentages of total variance explained by the axes are given in parentheses.

Of the 95 carbon sources, 40 showed no growth for any strain, seven allowed growth of all 153 non-S*higella E. coli* strains and 48 were variably used among the strains (Fig. 3, see also Fig. S1 for more details). On average, two strains differently used nine substrates. Thus, the growth capacities within the species were highly variable. The genetic diversity in *E. coli* species is highly structured (Escobar-Páramo *et al.*, 2004a). One hundred and fifty strains were classified into one of the four genetic groups (A/B1, B2, D and E). Each strain was also characterized by three lifestyles: its pathogenic group (commensal, ExPEC or InPEC), its host anthropogenic group (according to its exposure to humans: human, pet dog, farm animal or wildlife animal) and its host diet (insectivorous and granivorous bird, carnivorous mammal, herbivorous mammal or omnivorous mammal). To analyse the effect of the genetic group and the lifestyle on the growth yield, we carried out an anova for each carbon source allowing the growth of at least one strain. No significant effect was detected for the host anthropogenic group and the host diet, and only seven of the 55 substrates showed significant grouping effects (Table 1). For instance, the D-serine was differently used among the phylogroups. Members of the B2 group had a higher growth yield on average on this substrate than other strains, which confirmed the results obtained in a study using strains of serotype K1 mainly found in the B2 group (Moritz & Welch, 2006; Bidet *et al.*, 2007). On the contrary, the p-hydroxyphenylacetic acid was almost not used by the strains of the group B2 compared to other strains. Interestingly, the *hca* operon involved in the degradation of this substrate has been found specifically absent in all the group B2 strains (Touchon *et al.*, 2009). However, even in these cases, most of the variance remained unexplained by the model (*R*² ≤ 0.30). Consequently, on the plots of the PCA based on the growth yield, strains were not grouped by phylogroup or pathogenic group (Fig. 4) or any other lifestyle group (data not shown). Overall, the growth yield diversity did not structure the species into groups, as found previously (Fig. 2), as just a unique cloud of strains emerged from the PCA. Thus, the genetic group, as well as the lifestyle group we studied, were very weakly correlated to the growth yield, and, within a given group, phenotypes vary as much as across the whole non-*Shigella E. coli* strains.

**Table 1:** Significant grouping effects on the growth yield of 150 non-*Shigella Escherichia coli* strains in the analyses of variance accounting for genetic, pathogenic, host anthropogenic and host diet groups.

	Groups*						
				
Carbon sources	A/B1 (75)	B2 (38)	D (26)	E (11)	*F*-values†	*P*-values‡	*R*²
D-Galactonic acid lactone	0.82 (±0.03)	0.94 (±0.05)	1.01 (±0.06)	0.40 (±0.09)	11.58	8.12 × 10^−7^	0.23
D-Serine	0.40 (±0.04)	0.81 (±0.06)	0.40 (±0.07)	0.13 (±0.11)	12.50	2.79 × 10^−7^	0.22
Glycyl-L-aspartic acid	0.17 (±0.02)	0.35 (±0.03)	0.20 (±0.03)	0.18 (±0.05)	8.87	2.04 × 10^−5^	0.18
Lactulose	0.27 (±0.02)	0.22 (±0.03)	0.13 (±0.03)	0.10 (±0.05)	8.60	2.83 × 10^−5^	0.15
p-Hydroxyphenylacetic acid	0.52 (±0.04)	0.09 (±0.05)	0.35 (±0.07)	0.33 (±0.10)	13.15	1.33 × 10^−7^	0.30
		Commensal (87)	ExPEC (28)	InPEC (35)			
D,L-Lactic acid		1.10 (±0.02)	0.96 (±0.03)	1.00 (±0.02)	12.26	1.26 × 10^−5^	0.18
Uridine		0.51 (±0.02)	0.32 (±0.04)	0.35 (±0.03)	10.87	4.14 × 10^−5^	0.20

* Numbers of strains in the groups are indicated in parentheses next to the group label (the three ungrouped strains were discarded). For each carbon source with significant difference between groups, the least-square group mean is given, as well as its corresponding standard error in parentheses.

† The tested *F*-distributions had 3 and 138° of freedom for the genetic group effect and 2 and 138 for the pathogenic group effect.

‡ Only the effects for which the FDR was <0.1% were considered significant.

### Metabolic pathways are distributed according to the species phylogeny

**Fig. 5:**
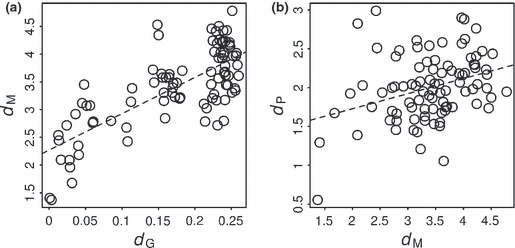
Relationships between the metabolic pathway completion distance, *d*_M_, and the genetic distance, *d*_G_, (a) and between the metabolic phenotypic distance, *d*_P_, and *d*_M_ (b) resulting from comparisons between 14 fully sequenced *E. coli* strains. The dashed lines correspond to the regression of *d*_M_ according to *d*_G_ (a) and *d*_P_ according to *d*_M_ (b).

To catabolize a carbon source, a strain must have specific enzymes. Consequently, to check whether the strain growth phenotypes reflect their metabolic gene content, we focused on 14 strains, which had their genome fully sequenced. We recovered the 395 metabolic pathways present in at least one of these 14 strains. About two-thirds (249 pathways) of these 395 pathways were conserved among the strains. A strong correlation (Mantel test *R*² = 0.56, *P*-value < 0.0001) was found between the genetic distance (*d*_G_) and the metabolic pathway completion distance (*d*_M_) (Fig. 5a). On the contrary, the correlation between *d*_P_ and *d*_M_ was weak (Mantel test *R*² = 0.11, *P*-value = 0.0057, Fig. 5b). Therefore, the presence/absence of metabolic pathways is linked to the genetic distance between strains and thus depends on their genetic group but is only weakly related to their growth yields.

To understand the weak correlation between *d*_P_ and *d*_M_, we tried to link the metabolic pathways to the carbon sources they catabolize. Of the 46 carbon sources allowing growth of at least one of the 14 sequenced strains, 43 were successfully linked to one or more pathways. For each strain-by-carbon source combination, we compared the growth status to the presence of the corresponding pathways. Overall, there was quite a good agreement between the presence or absence of metabolic pathways and the growth status as 73% of the cases were coherent, i.e. there was no growth when the pathway was absent or incomplete (53 cases) and growth when the pathway was complete (387 cases). However, we also found inconsistencies in 27 % of the cases, either strains growing while not having the complete required degradation pathway (42 cases) or strains not growing while having the complete degradation pathway (120 cases). Notice that the presence of the degradation pathway does not allow for quantitative predictions. For example, the cases for which the strains grew while not having the complete required degradation pathway did not correspond to particularly low growth yields as they varied in the same range as the growth yields resulting from complete pathways (data not shown).

### Few discrepancies between the metabolic pathways and the growth phenotypes are enough to decorrelate phenotypic and metabolic distances

**Fig. 6:**
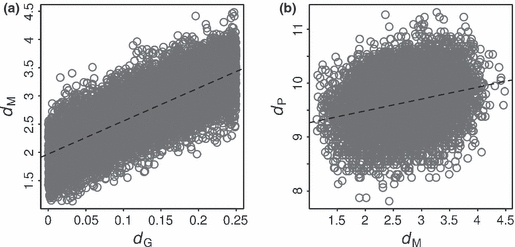
Relationships between metabolic pathway completion distance, *d*_M_, and genetic distance, *d*_G_, (a) and between metabolic phenotypic distance, *d*_P_, and *d*_M_ (b) resulting from the simulation of 10 000 strain pairs with a metabolic network composed of 395 pathways. The dashed lines correspond to the regression of *d*_M_ according to *d*_G_ (a) and *d*_P_ according to *d*_M_ (b).

To understand how *d*_M_ and *d*_P_ can be weakly correlated while the metabolic gene content explain most of the growth capacities, we modelled the phenotypes of a population of strains according to their metabolic gene presence and pathway functionality (all genes must be present for the pathway to be functional). The metabolic pathway completion distance is based on genome sequences and annotations and may not be fully indicative of the phenotypic distance. Indeed, two parameters translate the possible disruption between metabolic pathways and phenotypes. The first, *δ*_M_, is the probability that two strains share a common phenotype on a given substrate (growth or no growth) while having different pathway functionalities concerning this carbon source. The second, *δ*_P_, is the probability that two strains have different phenotypes emerging from the same pathway functionalities. Based on the discrepancies between the observed growth phenotypes and the predicted ones in our data set, we estimated on average *δ*_M_ = 0.63 and *δ*_P_ = 0.21. Interestingly, the high value for *δ*_M_ is mainly because of the cases where the two strains grew while having different pathway functionalities, which means that one strain could grow without the complete corresponding pathway. Using the relationship between the probability that two strains show different phenotypes on a given substrate and the probability that their related pathways have different functionality (eqn 3), we simulated the metabolic pathway distance and the metabolic phenotypic distance between 10 000 strain pairs with a metabolic network composed of 395 pathways (Fig. 6). The plot of the metabolic phenotypic distance according to the metabolic pathway distance thus obtained (Fig. 6b) was similar to the one experimentally observed (Fig. 5b). Moreover, the correlation between *d*_M_ and *d*_G_ (Fig. 6a) was indeed strong (*R*² = 0.58), as experimentally observed (Fig. 5a), whereas the one between *d*_P_ and *d*_M_ was weak (*R*² = 0.07). Therefore, the moderate proportion of discrepancies between the presence/absence of metabolic genes and growth phenotypes suffices to blur the link between metabolic phenotypic distance and metabolic pathway distance. The presence/absence of the metabolic pathways explains most of the growth capacities, that is the average strain metabolic phenotype, but is not a good predictor of the phenotypic differences between strains.

## Discussion

### The global phenotypic structure suggests continuous variations around an average behaviour within the species

We found that on average, in the Biolog GN2 microplate, a strain is able to metabolize 36 carbon sources, seven of which are common to all strains. Beside, several consumed substrates have been shown to be used in the natural habitats, such as L-arabinose, D-galactose, L-fucose, D-gluconic acid, N-acetyl-D-glucosamine, D-glucuronic acid and D-mannose (Chang *et al.*, 2004; Fabich *et al.*, 2008). We also found a great metabolic phenotypic diversity because between two strains nine substrates are differently used on average and globally 48 carbon sources could be used by some strains and not by others. This confirms that it is necessary to study several natural isolates to encompass more aspects of a ubiquitous species such as *E. coli* and that the laboratory model strain K-12 alone is definitely not representative of the whole species (Hobman *et al.*, 2007). The observed diversity is not surprising for microbial species as shown by previous numerical taxonomy studies (Johnson *et al.*, 1975; Sneath *et al.*, 1981). We assessed the effects of the strain phylogroup as well as of different lifestyles (pathogenicity, host exposure to humans and host diet) on the metabolic phenotypes, and we concluded that the metabolic phenotypic diversity of non-*Shigella E. coli* strains is very weakly linked to the strain phylogeny or to their lifestyle. Moreover, the observed variation is unlikely to be explained by other lifestyles as it did not appear to be structured at all. Indeed, the non-*Shigella E. coli* strain growth yield rather seems to present continuous variations around the species average.

### The metabolic phenotypes are versatile characters, quickly evolving

*In vivo*, *E. coli* has a mixed-substrate growth (Harder & Dijkhuizen, 1982; Lendenmann *et al.*, 1996). In environments that contain low concentrations of a variety of substrates, the ability to consume simultaneously several carbon sources even confers a competitive advantage. Indeed, the maximum growth rate of *E. coli* K-12 consuming simultaneously a mixture of two substrates is greater than its maximum growth rate when cultured with either one of the two carbon sources (Narang *et al.*, 1997). In addition, it has been shown that the ability to consume carbon sources impacts on *E. coli* colonization *in vivo* (Chang *et al.*, 2004). For several pathogens, specific metabolic capabilities constitute a fitness advantage or are even necessary for their spread, such as sucrose consumption for *Streptococcus pneumoniae* colonization (Iyer & Camilli, 2007) or lactate uptake for nasopharyngeal colonization by *Neisseria meningitidis* (Exley *et al.*, 2005). Moreover, in a new environment, metabolic capabilities of *E. coli* strains are optimized within a few hundred generations only (Dekel & Alon, 2005). Thus, being able to catabolize and use more than one carbon source is an advantage for both bacterial survival and spread. Therefore, a fraction of the observed metabolic phenotypic diversity might have been selected for and could be the result of the adaptation to slightly different environments. In this respect, the nutrient-niche hypothesis states that several ecological niches correspond to different nutrient availability within the intestine (Freter, 1983). In that case, the growth yield variation would correspond to different nutritional strategies of the strains adapted to continuous variations in their environment rather than to an environment compartmented into several discrete niches. Accordingly, EDL933 and K-12 have been shown to consume different carbon sources *in vivo* (Fabich *et al.*, 2008). Besides, the ecological niche of a strain is not constant because *E. coli* spends half of its life cycle in its primary habitat (gut of vertebrates) and the other half in the environment (water and soil) (Savageau, 1983). Its geographical spread is rapid and accompanied by frequent ecological niche shifts. For instance, in a farm environment, from the inoculation of a cow, a strain can be recovered from caretakers, mice, pigs, fowls and flies in a few days (Marshall *et al.*, 1990). Other studies showed that *E. coli* can establish and persist for a few days in fish intestines, giving them the opportunity to spread to distant waters (Rio-Rodriguez *et al.*, 1997; Guzmán *et al.*, 2004). Therefore, the continuous variations in metabolic phenotypes can also reflect the adaptation to past niches. Part of the large variability of metabolic phenotypes can also be neutral, having evolved by means of mutations, horizontal gene transfers and genetic drift. Indeed, the high mutational robustness of metabolic networks allows for phenotypic innovations at a low evolutionary cost, as it had been shown from *in silico* analyses (Matias Rodrigues & Wagner, 2009).

### Differences in regulatory networks can explain the disruption between genotypes and phenotypes

Less than half of the genome of a strain is shared by all the strains of the species (Rasko *et al.*, 2008; Touchon *et al.*, 2009). Consequently, one expects that part of the observed variation is because of unshared metabolic pathways obtained by horizontal gene transfers or differential gene loss. Accordingly, 73% of the diversity in growth capacities was explained by the presence/absence of degradation pathways. This proportion of explained growth is approximately the same as the level of agreement between experimental and computational results predicted by flux balance analysis calculations of a genome-scale metabolic reconstruction for *E. coli* K-12 (Feist *et al.*, 2007) and falls within the range found in published data on different microorganism species (between 57% and 94%) (Durot *et al.*, 2009). The agreement between growth and metabolic pathway presence in our data is relatively good considering that genome-scale models are more elaborate than our methodology, as they account for the network structure and are often refined with experimental data.

Although *E. coli* core genome represents only 11% of its pan-genome (Touchon *et al.*, 2009), we found that about two-thirds of the pathways present in the species show no difference in completion percentages between the 14 sequenced strains. This observation is in agreement with the fact that *E. coli* core metabolism represents 57% of its pan-metabolism (Vieira *et al.*, 2011). Moreover, 27% of the differences that we observed in metabolic capabilities were not explained by the presence or absence of the corresponding degradation pathways and were used to determine the parameters of the *in silico* simulations of metabolic phenotypes. Growth of strains that do not have the expected metabolic pathways can be caused presumably by unknown or unspecific enzymes that can catalyse several reactions. For instance, no strain had the complete pathway for the degradation of p-hydroxyphenylacetic acid as the enzyme catalysing one of its reactions is not described yet in the database. The cases for which strains had a metabolic pathway but did not grow on the corresponding carbon source can be because of mutations on coding genes or regulatory sequences, which can inactivate a metabolic pathway. Indeed, even if enzyme-coding genes are detected in a genome, missense mutations could still have modified the enzyme activity. Moreover, differences in regulatory networks between strains could affect the expression of the enzyme. For instance, it has been shown that the transcriptome was under selection in the *Shigella* strains (Le Gall *et al.*, 2005). In addition, the evolution of *E. coli* strains in laboratory conditions during a relatively short period revealed that most of the adaptation, i.e. increase in growth rate, was achieved by a transcriptional adjustment (Cooper *et al.*, 2003; Herring *et al.*, 2006). Likewise, the protein expression level has been shown to be rapidly optimized by evolution in *E. coli* (Dekel & Alon, 2005). On the whole, the presence/absence of metabolic pathways is a relatively good predictor of the average growth phenotype although there is an intermediate layer between the metabolic network and the phenotypes. Therefore, the metabolic pathways are distributed according to the phylogroups, but the discrepancies caused by the regulatory layer break this structure and lead to continuous variations in the metabolic phenotypes.

## Supporting information

**Figure S1** Diversity of carbon source use by 153 non-*Shigella E. coli* strains.

**Figure S2** Representation of the three Gaussian distributions that best fit the growth yield data according to the Bayesian information criterion (BIC) applied to Gaussian mixture models.

**Table S1** Characteristics of the *Escherichia* strains used in the study.

**Table S2** Metabolic pathways involved in the consumption of 43 carbon sources allowing growth of at least one of the 14 fully sequenced *E. coli* strains.

As a service to our authors and readers, this journal provides supporting information supplied by the authors. Such materials are peer-reviewed and may be reorganized for online delivery, but are not copy-edited or typeset. Technical support issues arising from supporting information (other than missing files) should be addressed to the authors.
