# Supplementary figures and images for: The decoupling between genetic structure and metabolic phenotypes in Escherichia coli leads to continuous phenotypic diversity
Source: J Evol Biol. 2011 Jul;24(7):1559–71. doi: 10.1111/j.1420-9101.2011.02287.x (PMC3147056; doi:10.1111/j.1420-9101.2011.02287.x)

# Supplementary figure

Fig. S1: Diversity of carbon source use by 153 non-*Shigella E. coli* strains.

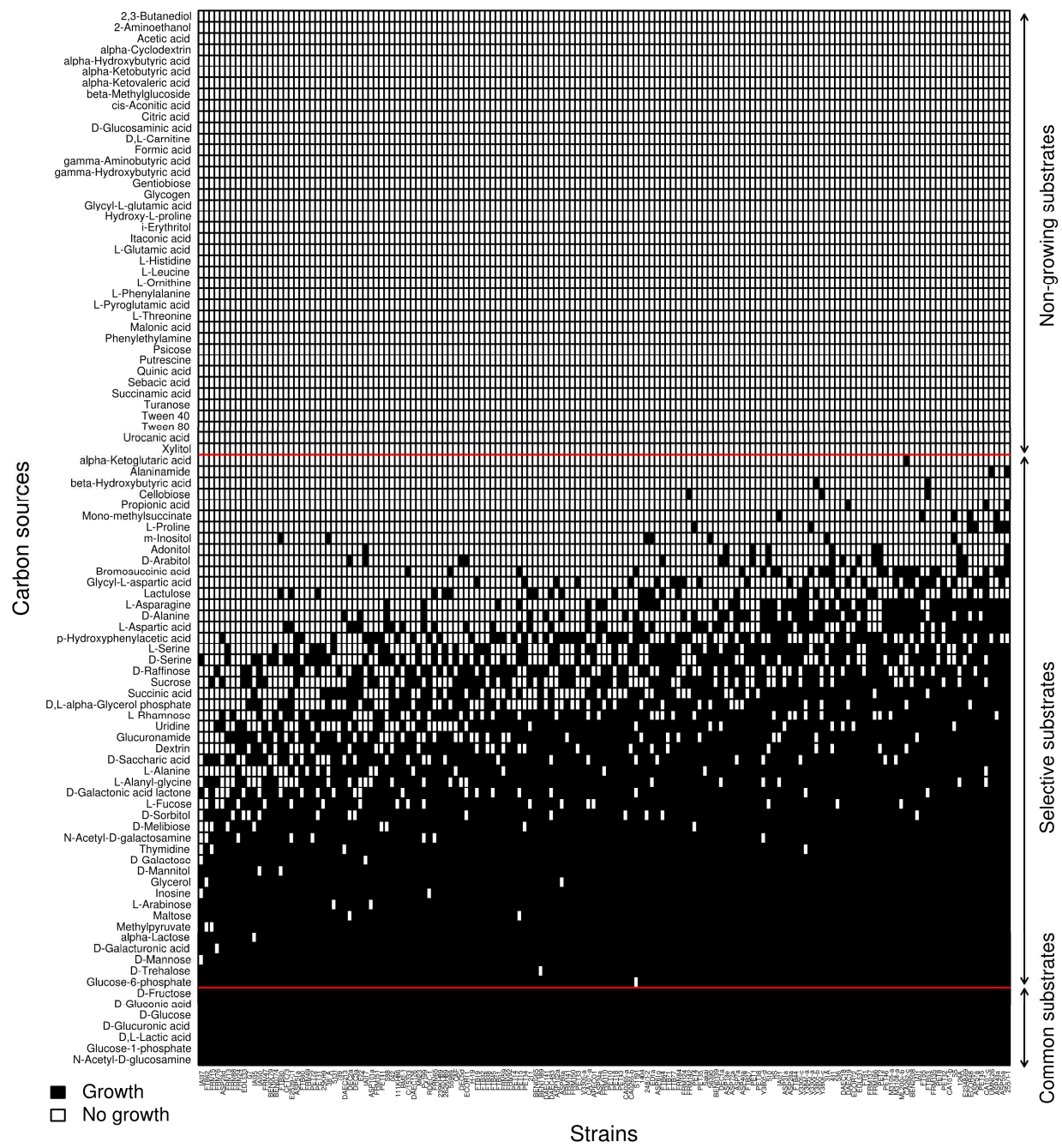

Supplement: Supplementary file 1 [file jeb0024-1559-SD1.pdf]
