# Supplementary material for: The decoupling between genetic structure and metabolic phenotypes in Escherichia coli leads to continuous phenotypic diversity
Source: J Evol Biol. 2011 Jul;24(7):1559–71. doi: 10.1111/j.1420-9101.2011.02287.x (PMC3147056; doi:10.1111/j.1420-9101.2011.02287.x)

Fig. S2: Representation of the three Gaussian distributions that best fit the growth yield data according to the Bayesian information criterion (BIC) applied to Gaussian mixture models.

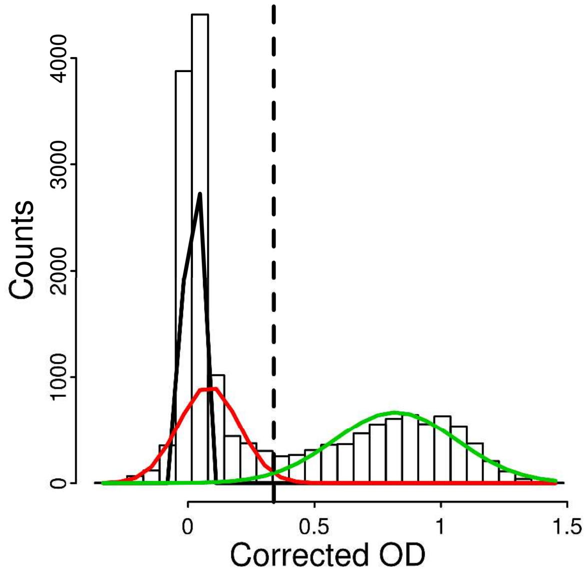

Supplement: Supplementary file 2 [file jeb0024-1559-SD2.pdf]
