# Supplementary material for: The decoupling between genetic structure and metabolic phenotypes in Escherichia coli leads to continuous phenotypic diversity
Source: J Evol Biol. 2011 Jul;24(7):1559–71. doi: 10.1111/j.1420-9101.2011.02287.x (PMC3147056; doi:10.1111/j.1420-9101.2011.02287.x)
Supplement: Supplementary file 3 [file jeb0024-1559-SD3.doc]

# Supplementary table

**Table S1: Characteristics of the *Escherichia*** strains used in the study.

| Strain ID | Host Linnean denomination | Host diet* | Host anthropogenic group | Genetic group^†^ | Pathogenic group^‡^ |
| --- | --- | --- | --- | --- | --- |
| 042 | *Homo sapiens sapiens* | On | Human | D | InPEC |
| 211 | *Bos taurus* | Hb | Farm | A/B1 | InPEC |
| 431 | *Sus scrofa* | On | Farm | A/B1 | InPEC |
| 510 | *Bos taurus* | Hb | Farm | A/B1 | InPEC |
| 536 | *Homo sapiens sapiens* | On | Human | B2 | ExPEC |
| 789 | *Gallus gallus* | Bd | Farm | A/B1 | ExPEC |
| 1404 | *Bos taurus* | Hb | Farm | A/B1 | ExPEC |
| 5131 | *Sus scrofa* | On | Farm | A/B1 | ExPEC |
| 55989 | *Homo sapiens sapiens* | On | Human | A/B1 | InPEC |
| 56390 | *Homo sapiens sapiens* | On | Human | D | InPEC |
| 111KH86 | *Bos taurus* | Hb | Farm | A/B1 | InPEC |
| 126A | *Bos taurus* | Hb | Farm | A/B1 | InPEC |
| 239KH89 | *Bos taurus* | Hb | Farm | A/B1 | ExPEC |
| 248/1-2 | *Bos taurus* | Hb | Farm | A/B1 | InPEC |
| 255/1-1 | *Bos taurus* | Hb | Farm | A/B1 | InPEC |
| 25KH9 | *Bos taurus* | Hb | Farm | A/B1 | InPEC |
| 262KH89 | *Bos taurus* | Hb | Farm | A/B1 | InPEC |
| 31A | *Bos taurus* | Hb | Farm | A/B1 | InPEC |
| 381A | *Homo sapiens sapiens* | On | Human | B2 | InPEC |
| APECO1 | *Gallus gallus* | Bd | Farm | B2 | ExPEC |
| ASP101a | *Lepus capensis* | Hb | Wildlife | A/B1 | Commensal |
| ASP102a | *Martes martes* | Cn | Wildlife | A/B1 | Commensal |
| ASP18e | *Ursus arctos* | Hb | Wildlife | A/B1 | Commensal |
| ASP28a | *Sus scrofa* | On | Wildlife | B2 | Commensal |
| ASP28d | *Sus scrofa* | On | Wildlife | A/B1 | Commensal |
| ASP31a | *Turdus merula* | Bd | Wildlife | D | Commensal |
| ASP34a | *Phasianus colchicus* | Bd | Wildlife | A/B1 | Commensal |
| ASP40a | *Pyrrhocorax pyrrhocorax* | Bd | Wildlife | E | Commensal |
| ASP43a | *Sus scrofa* | On | Wildlife | D | Commensal |
| ASP51a | *Vulpes vulpes* | Cn | Wildlife | D | Commensal |
| ASP55a | *Sus scrofa* | On | Wildlife | B2 | Commensal |
| ASP56e | *Cervus elaphus* | Hb | Wildlife | *Escherichia* clade V | Commensal |
| ASP57a | *Sus scrofa* | On | Wildlife | B2 | Commensal |
| ASP61g | *Martes martes* | Cn | Wildlife | B2 | Commensal |
| ASP72a | *Cervus elaphus* | Hb | Wildlife | A/B1 | Commensal |
| ASP73a | *Cervus elaphus* | Hb | Wildlife | *Escherichia* clade V | Commensal |
| ASP7a | *Cervus elaphus* | Hb | Wildlife | B2 | Commensal |
| ASP80a | *Cervus elaphus* | Hb | Wildlife | A/B1 | Commensal |
| BEN0079 | *Gallus gallus* | Bd | Farm | B2 | ExPEC |
| BEN0139 | *Meleagridis gallopavo* | Bd | Farm | B2 | ExPEC |
| BEN0265 | *Gallus gallus* | Bd | Farm | A/B1 | ExPEC |
| BEN0374 | *Gallus gallus* | Bd | Farm | B2 | ExPEC |
| BEN1189 | *Gallus gallus* | Bd | Farm | D | ExPEC |
| BEN2908 | *Gallus gallus* | Bd | Farm | B2 | ExPEC |
| BM2-1 | *Bos taurus* | Hb | Farm | B2 | ExPEC |
| C/15333 | *Bos taurus* | Hb | Farm | A/B1 | InPEC |
| CA0265-a | *Homo sapiens sapiens* | On | Human | A/B1 | Commensal |
| CA0265-b | *Homo sapiens sapiens* | On | Human | B2 | Commensal |
| CA107-a | *Homo sapiens sapiens* | On | Human | B2 | Commensal |
| CA107-b | *Homo sapiens sapiens* | On | Human | A/B1 | Commensal |
| CA107-c | *Homo sapiens sapiens* | On | Human | A/B1 | Commensal |
| CA262-c | *Homo sapiens sapiens* | On | Human | D | Commensal |
| CAF | *Homo sapiens sapiens* | On | Human | B2 | ExPEC |
| CFT073 | *Homo sapiens sapiens* | On | Human | B2 | ExPEC |
| CIP104947 | *Homo sapiens sapiens* | On | Human | *E. fergusonii* | Unknown |
| CIP107988 | *Homo sapiens sapiens* | On | Human | *E. albertii* | InPEC |
| DAEC126 | *Homo sapiens sapiens* | On | Human | A/B1 | InPEC |
| DAEC141 | *Homo sapiens sapiens* | On | Human | D | InPEC |
| DAEC18 | *Homo sapiens sapiens* | On | Human | B2 | InPEC |
| DAEC19 | *Homo sapiens sapiens* | On | Human | D | InPEC |
| DAEC213 | *Homo sapiens sapiens* | On | Human | D | InPEC |
| DAEC483 | *Homo sapiens sapiens* | On | Human | A/B1 | InPEC |
| DEC1a | *Homo sapiens sapiens* | On | Human | B2 | InPEC |
| DEC2a | *Homo sapiens sapiens* | On | Human | B2 | InPEC |
| DEC3a | *Homo sapiens sapiens* | On | Human | E | InPEC |
| DEC5d | *Homo sapiens sapiens* | On | Human | E | InPEC |
| E22 | *Oryctolagus cuniculus domestica* | Hb | Farm | A/B1 | InPEC |
| E2348/69 | *Homo sapiens sapiens* | On | Human | B2 | InPEC |
| E2539-C1 | *Homo sapiens sapiens* | On | Human | A/B1 | InPEC |
| ECOR11 | *Homo sapiens sapiens* | On | Human | A/B1 | ExPEC |
| ECOR48 | *Homo sapiens sapiens* | On | Human | D | ExPEC |
| ECOR72 | *Homo sapiens sapiens* | On | Human | A/B1 | ExPEC |
| ED1a | *Homo sapiens sapiens* | On | Human | B2 | Commensal |
| EDL931 | *Homo sapiens sapiens* | On | Human | E | InPEC |
| EDL933 | *Homo sapiens sapiens* | On | Human | E | InPEC |
| FRM110 | *Equus caballus* | Hb | Farm | D | Commensal |
| FRM124 | *Ovis aries* | Hb | Farm | A/B1 | Commensal |
| FRM13 | *Gallus gallus* | Bd | Farm | Ungrouped | Commensal |
| FRM135 | *Meleagris gallopavo* | Bd | Farm | B2 | Commensal |
| FRM137 | *Meleagris gallopavo* | Bd | Farm | A/B1 | Commensal |
| FRM141 | *Sus scrofa domestica* | On | Farm | A/B1 | Commensal |
| FRM142 | *Sus scrofa domestica* | On | Farm | E | Commensal |
| FRM15 | *Gallus gallus* | Bd | Farm | A/B1 | Commensal |
| FRM150 | *Sus scrofa domestica* | On | Farm | A/B1 | Commensal |
| FRM151 | *Oryctolagus cuniculus domestica* | Hb | Farm | A/B1 | Commensal |
| FRM166 | *Bos taurus* | Hb | Farm | B2 | Commensal |
| FRM24 | *Anser anser* | Bd | Farm | A/B1 | Commensal |
| FRM47 | *Bos taurus* | Hb | Farm | D | Commensal |
| FRM49 | *Oryctolagus cuniculus domestica* | Hb | Farm | B2 | Commensal |
| FRM59 | *Sus scrofa domestica* | On | Farm | A/B1 | Commensal |
| FRM65 | *Equus caballus* | Hb | Farm | A/B1 | Commensal |
| FRM76 | *Ovis aries* | Hb | Farm | E | Commensal |
| FRM84 | *Sus scrofa domestica* | On | Farm | A/B1 | Commensal |
| FRM88 | *Bos taurus* | Hb | Farm | A/B1 | Commensal |
| FRM92 | *Gallus gallus* | Bd | Farm | D | Commensal |
| FTB1 | *Capreolus capreolus* | Hb | Wildlife | A/B1 | Commensal |
| FTB19 | *Columba palumbus* | Bd | Wildlife | B2 | Commensal |
| FTB21 | *Sus scrofa* | On | Wildlife | *Escherichia* clade III | Commensal |
| FTB27 | *Cervus elaphus* | Hb | Wildlife | A/B1 | Commensal |
| FTB29 | *Martes martes* | Cn | Wildlife | *Escherichia* clade V | Commensal |
| FTB30 | *Sus scrofa* | On | Wildlife | E | Commensal |
| FTB32 | *Vulpes vulpes* | Cn | Wildlife | D | Commensal |
| FTB38 | *Oryctolagus cuniculus* | Hb | Wildlife | B2 | Commensal |
| FTB44 | *Cervus elaphus* | Hb | Wildlife | B2 | Commensal |
| FTB45 | *Cervus elaphus* | Hb | Wildlife | A/B1 | Commensal |
| FTB5 | *Columba palumbus* | Bd | Wildlife | B2 | Commensal |
| FTB51 | *Vulpes vulpes* | Cn | Wildlife | A/B1 | Commensal |
| FTB53 | *Oryctolagus cuniculus* | Hb | Wildlife | Ungrouped | Commensal |
| FTB62 | *Cervus elaphus* | Hb | Wildlife | A/B1 | Commensal |
| FTB64 | *Capreolus capreolus* | Hb | Wildlife | B2 | Commensal |
| FTB71 | *Capreolus capreolus* | Hb | Wildlife | A/B1 | Commensal |
| FTB74 | *Sus scrofa* | On | Wildlife | *Escherichia* clade III | Commensal |
| FTB77 | *Sus scrofa* | On | Wildlife | B2 | Commensal |
| FTB80 | *Sus scrofa* | On | Wildlife | A/B1 | Commensal |
| G7 | *Sus scrofa* | On | Farm | A/B1 | InPEC |
| H-19 | *Homo sapiens sapiens* | On | Human | A/B1 | InPEC |
| HS | *Homo sapiens sapiens* | On | Human | A/B1 | Commensal |
| IAI1 | *Homo sapiens sapiens* | On | Human | A/B1 | Commensal |
| IAI17 | *Homo sapiens sapiens* | On | Human | A/B1 | ExPEC |
| IAI21 | *Homo sapiens sapiens* | On | Human | A/B1 | ExPEC |
| IAI35 | *Homo sapiens sapiens* | On | Human | D | ExPEC |
| IAI37 | *Homo sapiens sapiens* | On | Human | A/B1 | ExPEC |
| IAI50 | *Homo sapiens sapiens* | On | Human | E | ExPEC |
| K-12 | *Homo sapiens sapiens* | On | Lab | A/B1 | Commensal |
| M3105-a | *Homo sapiens sapiens* | On | Human | D | Commensal |
| M3108-a | *Homo sapiens sapiens* | On | Human | B2 | Commensal |
| M623 | *Sus scrofa* | On | Farm | B2 | ExPEC |
| ML2004-b | *Homo sapiens sapiens* | On | Human | A/B1 | Commensal |
| PET1 | *Canis familiaris* | Cn | Pet | A/B1 | Commensal |
| PET10 | *Canis familiaris* | Cn | Pet | A/B1 | Commensal |
| PET14 | *Canis familiaris* | Cn | Pet | D | Commensal |
| PET15 | *Canis familiaris* | Cn | Pet | A/B1 | Commensal |
| PET18 | *Canis familiaris* | Cn | Pet | A/B1 | Commensal |
| PET2 | *Canis familiaris* | Cn | Pet | A/B1 | Commensal |
| PET22 | *Canis familiaris* | Cn | Pet | A/B1 | Commensal |
| PET31 | *Canis familiaris* | Cn | Pet | A/B1 | Commensal |
| PET32 | *Canis familiaris* | Cn | Pet | A/B1 | Commensal |
| PET33 | *Canis familiaris* | Cn | Pet | D | Commensal |
| PET34 | *Canis familiaris* | Cn | Pet | D | Commensal |
| PET35 | *Canis familiaris* | Cn | Pet | B2 | Commensal |
| PET36 | *Canis familiaris* | Cn | Pet | *Escherichia* clade V | Commensal |
| PET38 | *Canis familiaris* | Cn | Pet | A/B1 | Commensal |
| PET4 | *Canis familiaris* | Cn | Pet | B2 | Commensal |
| PET41 | *Canis familiaris* | Cn | Pet | D | Commensal |
| PET43 | *Canis familiaris* | Cn | Pet | A/B1 | Commensal |
| PET45 | *Canis familiaris* | Cn | Pet | B2 | Commensal |
| PET46 | *Canis familiaris* | Cn | Pet | D | Commensal |
| PET8 | *Canis familiaris* | Cn | Pet | A/B1 | Commensal |
| Py199 | *Homo sapiens sapiens* | On | Human | D | ExPEC |
| RDEC-1 | *Oryctolagus cuniculus domestica* | Hb | Farm | A/B1 | InPEC |
| ROAR344 | *Homo sapiens sapiens* | On | Human | *E. fergusonii* | Commensal |
| S1191 | *Sus scrofa* | On | Farm | A/B1 | InPEC |
| S5 | *Ovis aries* | Hb | Farm | A/B1 | ExPEC |
| S88 | *Homo sapiens sapiens* | On | Human | B2 | ExPEC |
| Sakai | *Homo sapiens sapiens* | On | Human | E | InPEC |
| SB01-97 | *Homo sapiens sapiens* | On | Human | S1 | *Shigella* |
| SB11-56 | *Homo sapiens sapiens* | On | Human | S2 | *Shigella* |
| SD01-55 | *Homo sapiens sapiens* | On | Human | SD1 | *Shigella* |
| SD05-73 | *Homo sapiens sapiens* | On | Human | S1 | *Shigella* |
| SF04-92 | *Homo sapiens sapiens* | On | Human | S3 | *Shigella* |
| SS94a | *Homo sapiens sapiens* | On | Human | SS | *Shigella* |
| UMN026 | *Homo sapiens sapiens* | On | Human | D | ExPEC |
| V3306-a | *Homo sapiens sapiens* | On | Human | D | Commensal |
| V3309-a | *Homo sapiens sapiens* | On | Human | A/B1 | Commensal |
| V3309-c | *Homo sapiens sapiens* | On | Human | B2 | Commensal |
| Y3805-b | *Homo sapiens sapiens* | On | Human | A/B1 | Commensal |
| Y3805-d | *Homo sapiens sapiens* | On | Human | D | Commensal |
| Y3806-c | *Homo sapiens sapiens* | On | Human | Ungrouped | Commensal |
| Y3840-a | *Homo sapiens sapiens* | On | Human | E | Commensal |
| *Bd, insectivorous and granivorous birds; Cn, carnivorous mammals; Hb, herbivorous mammals; On, omnivorous mammals. | | | | | |
| ^†^Genetic groups were defined using the MLST data. | | | | | |
| ^‡^InPEC (intestinal pathogenic *E. coli*), ExPEC (extra-intestinal pathogenic *E. coli*). | | | | | |

**Table S2: Metabolic pathways involved in the consumption of 43 carbon sources allowing growth of at least one of the 14 fully sequenced *E. coli*** strains.

| Carbon source | Metabolic pathway* |
| --- | --- |
| D-Glucose | glycolysis I |
| D-Glucose | pentose phosphate pathway (oxidative branch) |
| alpha-Ketoglutaric acid | 2-ketoglutarate dehydrogenase complex |
| alpha-Lactose | lactose degradation II |
| alpha-Lactose | lactose degradation III |
| D-Alanine | alanine degradation I |
| D-Arabitol | D-arabitol degradation |
| D-Fructose | fructose degradation |
| D-Galactonic acid lactone | D-galactonate degradation |
| D-Galactose | galactose degradation I (Leloir pathway) |
| D-Galacturonic acid | D-galacturonate degradation |
| D-Gluconic acid | superpathway of gluconate degradation |
| D-Glucuronic acid | beta-D-glucuronide and D-glucuronate degradation |
| D-Mannitol | mannitol degradation I |
| D-Mannose | D-mannose degradation |
| D-Melibiose | melibiose degradation |
| D-Raffinose | D-raffinose degradation^†^ |
| D-Saccharic acid | D-glucarate degradation |
| D-Serine | D-serine degradation^†^ |
| D-Sorbitol | sorbitol degradation I |
| D-Sorbitol | sorbitol degradation II |
| D-Trehalose | trehalose degradation I (low osmolarity) |
| D-Trehalose | trehalose degradation II (trehalase) |
| D,L-alpha-Glycerol phosphate | glycerol degradation I |
| D,L-Lactic acid | L-lactaldehyde degradation (aerobic) |
| Dextrin | glycogen degradation I |
| Glucose-1-phosphate | glucose and glucose-1-phosphate degradation |
| Glucose-6-phosphate | glycolysis I |
| Glucose-6-phosphate | pentose phosphate pathway (oxidative branch) |
| Glycerol | glycerol degradation I |
| Glycerol | glycerol degradation III |
| Glycerol | glycerol degradation V |
| Inosine | degradation of purine ribonucleosides |
| L-Alanine | alanine degradation I |
| L-Arabinose | L-arabinose degradation I |
| L-Asparagine | asparagine degradation I |
| L-Aspartic acid | aspartate degradation II |
| L-Fucose | fucose degradation |
| L-Rhamnose | rhamnose degradation |
| L-Serine | L-serine degradation |
| Lactulose | lactulose degradation^†^ |
| m-Inositol | myo-inositol degradation |
| Maltose | glycogen degradation I |
| N-Acetyl-D-galactosamine | N-acetyl-D-galactosamine degradation^†^ |
| N-Acetyl-D-glucosamine | N-acetylglucosamine degradation |
| p-Hydroxyphenylacetic acid | 4-hydroxyphenylacetate degradation |
| Succinic acid | succinate to cytochrome bd oxidase electron transfer |
| Succinic acid | succinate to cytochrome bo oxidase electron transfer |
| Sucrose | sucrose degradation I |
| Thymidine | pyrimidine deoxyribonucleosides degradation |
| Uridine | degradation of pyrimidine ribonucleosides |
| *The metabolic pathways were recovered from the Metacyc database (http://metacyc.org). | |
| ^†^Manually added pathways. | |


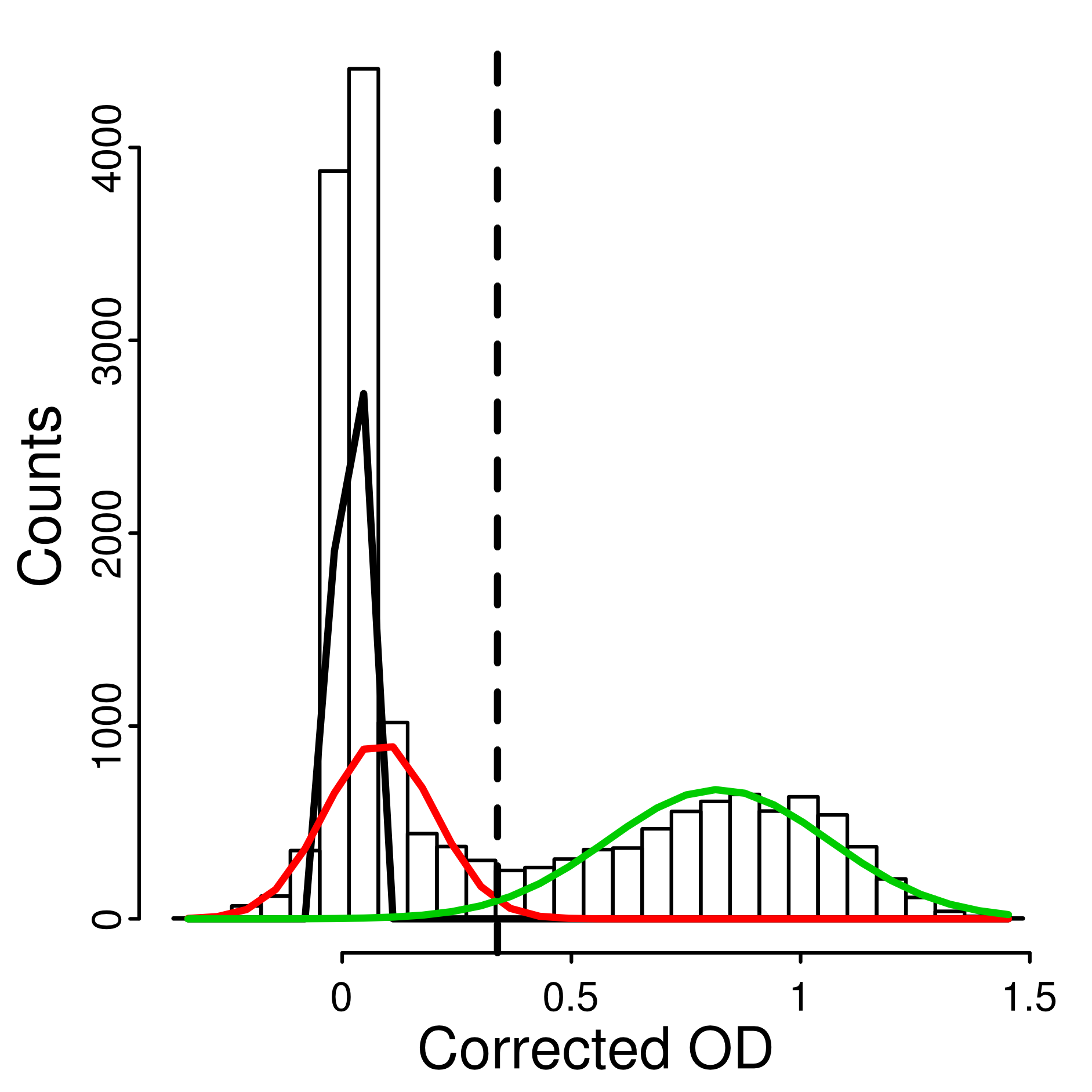


# Supplementary figure legends

**Fig. S1: Diversity of carbon source use by 153 non-*Shigella* *E. coli* strains.** Seven carbon sources allowed the growth of all the 153 strains (common substrates) and 48 of only a fraction of them (selective substrates) whereas 40 did not allow any growth (nongrowing substrates). The strains are ordered by the number of substrates they can catabolise. The carbon sources are ordered by the number of strains able to grow on them.

**Fig. S2: Representation of the three Gaussian distributions that best fit the growth yield data according to the Bayesian information criterion (BIC) applied to Gaussian mixture models.** The vertical dashed line represents the threshold (0.3388 OD units) that separates the two first components from the third one and above which growth was considered to be positive.
